# Whole proteome mapping of compound-protein interactions

**DOI:** 10.1016/j.crchbi.2022.100035

**Published:** 2022-09-12

**Authors:** Venkat R. Chirasani, Jian Wang, Congzhou Sha, Wesley Raup-Konsavage, Kent Vrana, Nikolay V. Dokholyan

**Affiliations:** a Department of Pharmacology, Penn State College of Medicine, Hershey, PA, 17033, USA; b Department of Biochemistry and Biophysics, University of North Carolina at Chapel Hill, Chapel Hill, NC, 27599, USA; c Department of Biochemistry & Molecular Biology, Penn State College of Medicine, Hershey, PA, 17033, USA; d Department of Chemistry, Pennsylvania State University, University Park, PA, 16802, USA; e Department of Biomedical Engineering, Pennsylvania State University, University Park, PA, 16802, USA

**Keywords:** Chemical similarity, Neural network, Target identification, Polypharmacology, Cannabigerol

## Abstract

Off-target binding is one of the primary causes of toxic side effects of drugs in clinical development, resulting in failures of clinical trials. While off-target drug binding is a known phenomenon, experimental identification of the undesired protein binders can be prohibitively expensive due to the large pool of possible biological targets. Here, we propose a new strategy combining chemical similarity principle and deep learning to enable proteome-wide mapping of compound-protein interactions. We have developed a pipeline to identify the targets of bioactive molecules by matching them with chemically similar annotated “bait” compounds and ranking them with deep learning. We have constructed a user-friendly web server for drug-target identification based on chemical similarity (DRIFT) to perform searches across annotated bioactive compound datasets, thus enabling high-throughput, multi-ligand target identification, as well as chemical fragmentation of target-binding moieties.

## 1. Introduction

Drug discovery and development is a slow and expensive process (Dickson and Gagnon, 2009). Developing a new medicine through FDA approval takes approximately 10 years and costs, on average, $2.6 billion (DiMasi et al., 2016). Apart from the long and expensive drug development timeline, approved drugs often suffer from poor activity (rare interaction with intended protein target) and high side-effect profiles (binding with other targets) (Xie et al., 2012). As the rule, rather than the exception, drug molecules in living cells bind to myriad structurally similar target proteins in addition to the intended target. The interaction of drugs with unintended targets (off-targets) may generate adverse drug reactions (Bowes et al., 2012; Campillos et al., 2008; Roth, 2007), such as the severe side effects induced by the fatty acid amide hydrolase (FAAH) inhibitor BIA 10–2474 in a recent clinical trial (Hayes et al., 2021). By predicting a side effect profile and potential off-targets during the preclinical discovery phase, we may be able to reduce drug failure rate during clinical studies and expedite the drug discovery process. On the other hand, modulating multiple targets may be therapeutically beneficial in diseases such as cancer, neuropsychiatric disorders, and infectious diseases (Brotz-Oesterhelt and Brunner, 2008; Knight et al., 2010; Roth et al., 2004). Decrypting the target interaction space of a drug or lead compound is important for determining its safety, efficacy, and potential for repurposing. Characterizing the multi-target profile of specific a drug molecule – either to reduce off-target binding or to achieve a desired polypharmacological effect – is currently a daunting task for medicinal chemists.

Due to their speed and accuracy, *in silico* target prediction methods play a significant role in estimating the protein targets and pharmacological properties of chemical compounds (Schenone et al., 2013; Terstappen et al., 2007), and can be classified into two categories: (1) structure-based and (2) ligand-based methods. Structure-based target prediction methods, such as TarFisDock (Li et al., 2006), MedusaDock (Ding et al., 2010; Ding and Dokholyan, 2013; Wang and Dokholyan, 2019), and INVDOCK (Chen and Zhi, 2001) explore the target interaction space of active chemical compounds through molecular docking, whereby a drug is positioned through simulations inside the binding pocket of the target and the free energy or another scoring parameter is used to evaluate the strength of the binding interaction. Due to the high computational cost of molecular docking and need for high-resolution protein 3D structures, wide-scale adoption and applications of these structure-based methods have been limited. In contrast, ligand-based prediction methods, such as HitPick, SuperPred, ChemMapper, similarity ensemble approach (SEA), TargetHunter, CSNAP3D, and SwissTargetPrediction (Daina et al., 2019; Dunkel et al., 2008; Gong et al., 2013; Keiser et al., 2007; Lo et al., 2015; Wang et al., 2013) are computationally inexpensive. However, these ligand-based prediction methods often fail to predict the target interaction space for new chemical compounds with low structural similarity to known drug molecules. Additionally, the rapid growth of annotated chemical databases requires an advanced computational strategy to efficiently quantify structural similarity between query and annotated compounds. To this end, we have previously developed methodologies to estimate off-target binding by screening the entire RCSB database for a given substructure (Shirvanyants et al., 2011) or for proteins that have local surface similarities (Yin et al., 2009). These methodologies are limited to available high-resolution protein 3D structures deposited to a protein database (Berman et al., 2000).

**Fig. 1.**
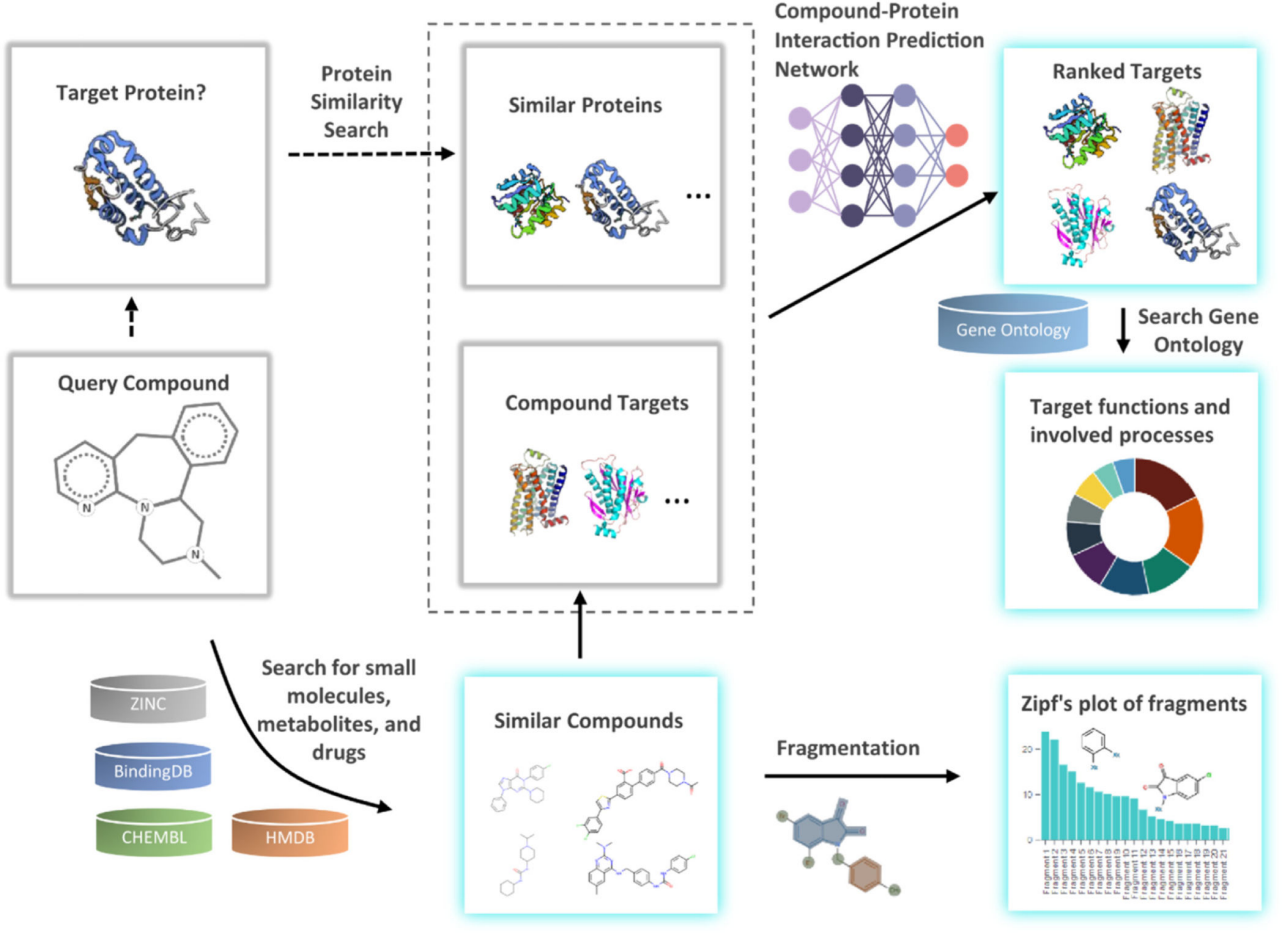
Flow chart outlining the processing steps of compound targets identification in DRIFT. Given the query compound, DRIFT first searches against ZINC, BindingDB, CHEMBL, and HMDB for small molecules, metabolites, and drugs that are similar to the query compound based on FP2 fingerprints and 3D pharmacophores. DRIFT then searches for targets associated with these bait compounds. These compounds are fragmented to identify the fragments that play key roles in binding with the targets. These similar targets and the targets of the bait compounds are then subject to the DeepDrift neural network to find the most likely targets. Finally, DRIFT identifies the functions and involved processes for each target. Highlighted cards refer to outputs of DRIFT webserver.

Capitalizing on our recent successes in formulating strategies for protein design (Yin et al., 2007; Zhu et al., 2017, 2019), protein-ligand docking (Ding et al., 2010; Ding and Dokholyan, 2013), and RNA design (Wang et al., 2019), we devise a new pipeline, DRIFT (Fig. 1), that enables proteome-wide identification of protein binders (both on-target and off-target) of any active chemical molecule. DRIFT searches for compounds based on both 2D and 3D similarities and then extracts their associated targets. We constructed an attention-based neural network to rank identified targets based on the extent of compound-protein interactions. We herein evaluate the performance of DRIFT in terms of similar compound searching (Böhm et al., 2004), compound-protein interaction prediction, and drug target identification using three independent datasets. We provide a user-friendly webserver, DRIFT (http://Drift.Dokhlab.org), to facilitate the use of our pipeline for drug target identification. Finally, we utilized DRIFT to analyze the target interaction space of doxorubicin, serotonin, β-endorphin, and cannabigerol. Based on the drug-target interaction space estimated by DRIFT, we can iteratively modify any annotated drug molecule to improve its pharmaceutical properties and subsequently minimize off-target interactions. Our computational strategy may assist in designing safer and more effective medications, while reducing costs in traditional target identification methodologies.

## 2. Results

### 2.1. Comprehensive evaluation of DRIFT’s performance

Most target identification methods are based on similarity property principle. Given a query compound, they identify chemically similar compounds and then hypothesize that the targets of these identified similar compounds may also bind to the query compound. DRIFT is also based on similarity property principle, and in addition, DRIFT uses an attention-based neural network (Wang and Dokholyan, 2022) that we developed previously to predict compound-protein interaction to rank all protein targets of the compounds that have been identified based on chemical similarity. Thus, DRIFT is mainly composed of two components: a compound-searching component, which is responsible for identifying chemically similar compounds based on the query compound, and a target-ranking component, which is responsible for ranking the targets to select the ones that have the highest binding probability to the query compound.

**Fig. 2.**
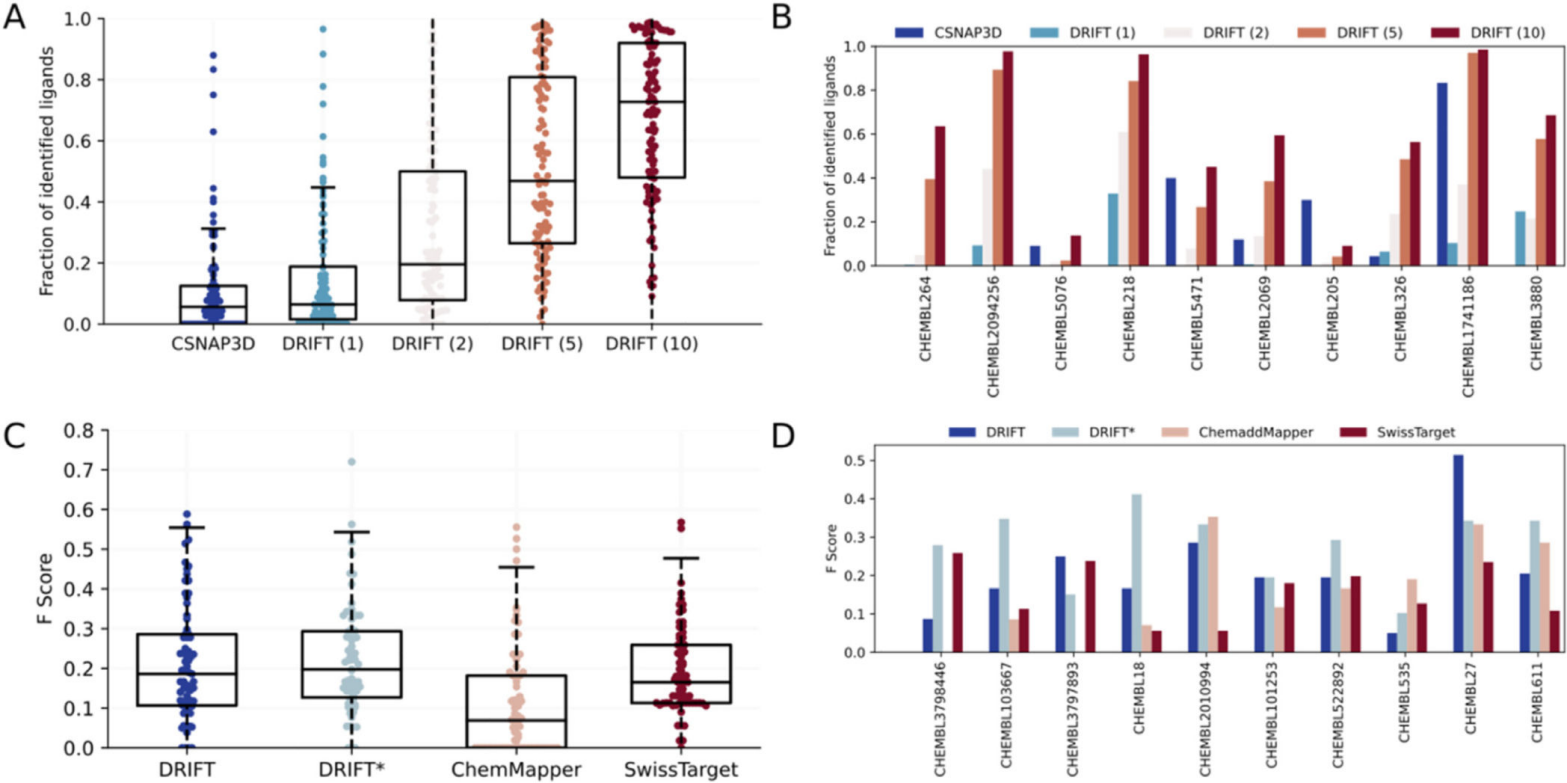
Comprehensive evaluation of DRIFT’s performance. (A) The fraction of the compounds identified by DRIFT and CSNAP3D in DS-I. DRIFT (1), DRIFT (2), DRIFT (5), and DRIFT (10) represent the results of DRIFT by using 1, 2, 5, and 10 3D conformers in the pharmacophore search. (B) The fraction of compounds identified by DRIFT and CSNAP3D for 10 of the proteins in DS-I. (C) The F Score of identifying targets by DRIFT, ChemMapper, and SwissTargetPrediction in DS-II. DRIFT* refers to the results of DRIFT by excluding the direct interactions between the query compound and its associated targets from the database. 10 conformers were used in DRIFT’s pharmacophore search. (D) The F Score of identifying targets for 10 of the compounds in DS-II.

The compound-searching component of DRIFT is based on both 2D and 3D similarities of compounds. The 2D similarity is evaluated by FP2 fingerprint, a typical path-based fingerprint which indexes small molecule fragments based on linear segments of up to 7 atoms; the 3D similarity is evaluated by pharmacophore (see Methods). We first compare DRIFT to CSNAP3D to analyze the ability (Böhm et al., 2004) to identify similar compounds. We compile a dataset (DS-I) composed of 109 proteins, each of which has at least 20 compounds. For each protein, we select one of its compounds as the query compound and use DRIFT to identify similar compounds. We test the performance of DRIFT by using different numbers (1, 2, 5, and 10) of 3D conformers for pharmacophore search. We calculate the fraction of the associated compounds that are successfully identified by DRIFT and compare the result to CSNAP3D. CSNAP3D does not support 3D similarity search. Averagely, CSNAP3D can only identify 11.1% of the compounds. DRIFT can identify 15.5%, 29.9%, 52.2%, or 67.6% of the compounds if 1, 2, 5, or 10 conformers are used in pharmacophore search (Fig. 2A & B). Thus, we used 10 conformers for the pharmacophore search in the final DRIFT pipeline. Of note, we have tested the fraction of identified ligands, which is the recall, but we did not test the precision. This is because the false positives will be taken into consideration in the next step, target identification. In this step, we would only like to find as many true positives as possible.

The target-ranking component of DRIFT is based on an attention-based neural network, Yuel (Wang and Dokholyan, 2022), that we have developed previously to evaluate compound-protein interaction based on the 2D structure of the compound and the sequence of the protein. Compared to other sequence-based compound-protein interaction prediction neural networks, Yuel circumvents the overfitting issue by using feature-wise fully connected layers and attention. We have trained and tested Yuel and two other representative sequence-based compound-protein interaction prediction neural networks, DeepDTA (Öztürk et al., 2018) and DeepConv-DTI (Lee et al., 2019), on two data sets, PDBbind (Liu et al., 2015) and Davis (Davis et al., 2011). We have divided each of the two data sets into a training set and a test set at a ratio of 8 to 2, resulting in four data sets (Davis/train, Davis/test, PDBbind/-train, PDBbind/test). By training on Davis/train, the Pearson correlation coefficients between the experimental affinities and predicted affinities of Yuel, DeepDTA, and DeepConv-DTI on Davis/test are all around 0.6; by training on PDBbind/train, the Pearson correlation coefficients on PDBbind/test are all around 0.7. However, when training on Davis/train and testing on PDBbind/test, the Pearson correlation coefficients of Yuel, DeepDTA, and DeepConv-DTI are 0.46, 0.10, and 0.08, suggesting that DeepDTA, and DeepConv-DTI cannot predict the interaction between unknown proteins and unknown compounds (there is a very low similarity between PDBbind and Davis), while Yuel can predict many of these interactions.

Finally, we evaluate the ability of DRIFT to predict off-targets. We first evaluate DRIFT on a dataset (Davis et al., 2011) composed of 72 kinase inhibitors with 442 kinases covering >80% of the human catalytic protein kinome and including different kinase families. As shown in Fig. S1, we have analyzed the ROC (Receiver Operating Characteristic curve) to evaluate both the precision and the false positive rates. The AUC (Area Under the ROC Curve) is 0.86, indicating that DRIFT has both low false positive rate and high true positive rate. Next, we compile another dataset consisting of 110 compounds and their 2323 protein targets (DS-II) to evaluate the ability of DRIFT to identify off-targets. In this dataset, each compound is associated with at least 5 proteins. For each compound, we first determine the fraction of targets that are correctly predicted, i.e. the recall. We compare DRIFT to two other target identification methods ChemMapper and SwissTargetPrediction. The average recall of DRIFT, ChemMapper, and SwissTargetPrediction are 0.97, 0.15, and 0.79, respectively. To consider the false positives, we calculate the F-score (Fig. 2C and D). The average F-score of DRIFT, ChemMapper, and SwissTargetPrediction are 0.21, 0.12, and 0.19, respectively. DRIFT outperforms ChemMapper and SwissTargetPrediction, demonstrating DRIFT’s higher ability to identify drug targets. Compared to the recall, the F score is low, and this may be reasoned at two aspects. First, there are some false positives in the predicted targets; second, some of the predicted targets may be real targets but have not been validated experimentally. Of note, when testing the ability of these methods to identify targets, many of the compound-protein pairs in the test set have already been deposited in the database used for similar compounds search. To evaluate the ability to find unknown targets, we exclude the interactions between the query compound and its own experimentally validated targets. In this case, the F score of DRIFT is 0.22 (Fig. 2C), without any performance decrease. Since SwissTargetPrediction and ChemMapper only provide webservers, we were unable to test their ability to find unknown targets. Overall, the low F scores of all these methods indicate that more methods and more target screening data are needed to change the *status quo* of the target identification field.

### 2.2. DRIFT predicts targets of doxorubicin

**Fig. 3.**
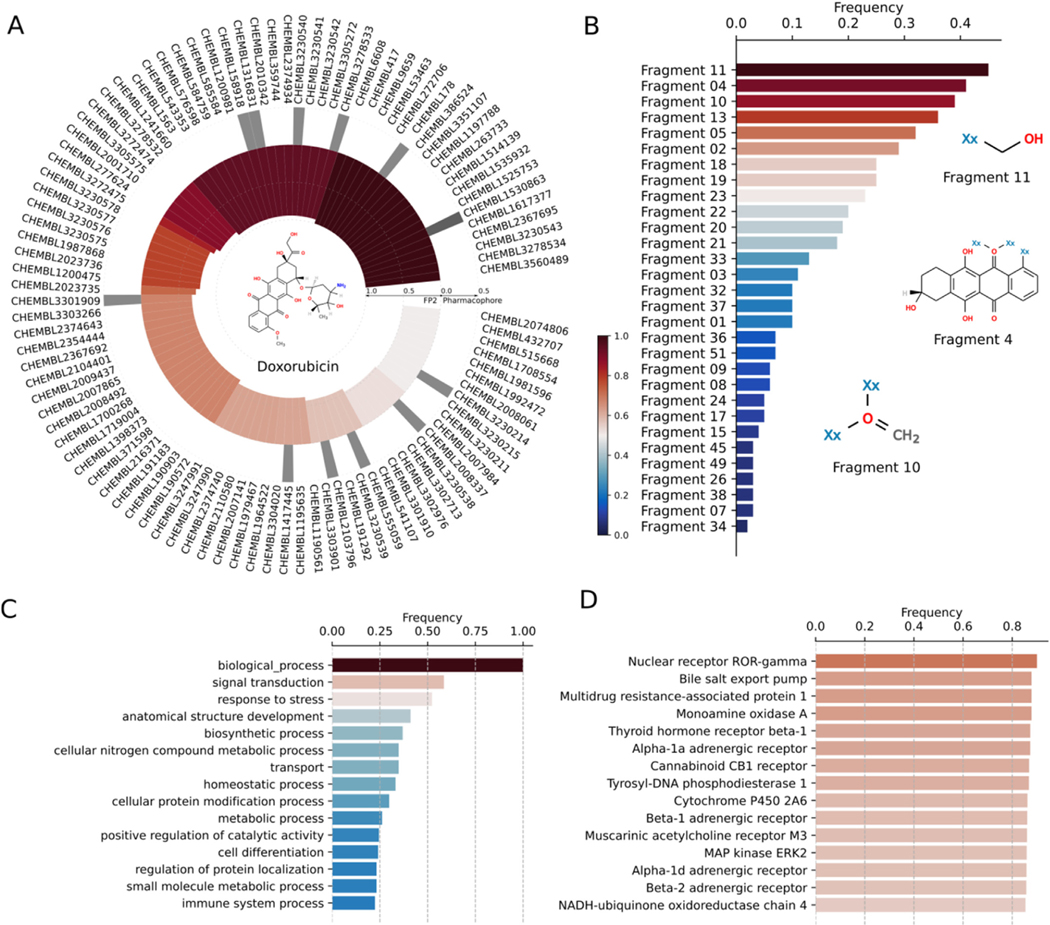
(A) Circular plot depicting the ChEMBL IDs of doxorubicin’s bait compounds. 2D structures of bait compounds is included in the center of the circular plot. The outer bars refer to the extent of pharmacophore similarity of compounds and the inner bars refer to the extent of FP2 similarity of compounds. (B) Zipf’s plot showing the rank distribution of chemical fragments of doxorubicin with respect to their frequency of occurrence. 2D structures of three most frequently occurring fragments of doxorubicin and its bait compounds are shown. (C) Top 15 biological processes or functions executed by the targets of Doxorubicin and its bait compounds. (D) The list of top 15 biological targets of Doxorubicin as estimated by DRIFT.

We employ DRIFT to predict targets of doxorubicin, a frequently used chemotherapeutic anthracycline anticancer drug with a broad side-effect profile due to numerous off-target interactions (Jordon, 2002; Weiss, 1992). Doxorubicin inhibits the activity of topoisomerase-II to prevent the growth of cancer cells (Pommier et al., 2010). Specifically, doxorubicin terminates the process of DNA replication by preventing the topoisomerase-II enzyme from resealing the broken DNA double helix after cleavage and unwinding of the DNA. Doxorubicin is known to induce severe toxicological side effects. Common effects of doxorubicin are vomiting, hair loss, skin rashes, and mouth inflammation, which are presumably due to the interaction of doxorubicin with multiple target proteins. One of the major side effects of doxorubicin is dilated cardiomyopathy (Chatterjee et al., 2010), which causes congestive heart failure. The incidence of cardiotoxicity during doxorubicin treatment is acute and can occur 2–3 days after the administration of doxorubicin during chemotherapy. Clinical studies have identified a few off-targets of doxorubicin, which may trigger the above-mentioned side effects. Specifically, doxorubicin has high affinity towards mitochondrial enzymes such as xanthine oxidase, NADH dehydrogenase, and cytochrome P450 reductase to generate reactive oxygen species during dilated cardiomyopathy (Bachur et al., 1979; Berlin and Haseltine, 1981; Davies and Doroshow, 1986; Takemura and Fujiwara, 2007). Given the severity of doxorubicin-induced cardiotoxicity and other side effects, the lack of knowledge of the target interaction space of doxorubicin may limit its usage during chemotherapy. A detailed interaction map of doxorubicin may enable the design of doxorubicin-related compounds to reduce these adverse effects. We employed DRIFT to identify and characterize a wide range of off-targets of doxorubicin (Fig. 3). We perform a chemical similarity search for doxorubicin against ChEMBL (Gaulton et al., 2011), HMDB (Wishart et al., 2018), BindingDB (Wishart et al., 2017) and Zinc (Irwin et al., 2012) databases using DRIFT. Interestingly, our chemical similarity search approach identified nuclear receptor ROR-gamma and tyrosyl-DNA phosphodiesterase-1, which regulate the DNA transcription process, as potential targets of Doxorubicin (Fig. 3D). Additionally, DRIFT identified monoamine oxidase A and cytochrome P-450 reductase, which play a potential role in cardiomyocyte damage and pathogenesis of heart failure, as potential targets of doxorubicin. Furthermore, the off-target prediction process utilizing our computational strategy corroborates experimental data by identifying mitogen-activated protein kinase 1/extracellular signal-regulated kinase-2 (MAPK1/ERK2) as a potential off-target of doxorubicin (Lou et al., 2005). Apart from the annotated targets of doxorubicin, DRIFT also identified several other off-targets of doxorubicin, such as multidrug resistance-associated protein 1, thyroid hormone receptor beta-1, adrenergic receptor-α1A, β1, cannabinoid CB1 receptor, and muscarinic acetylcholine receptor-M3 (Fig. 3D).

### 2.3. DRIFT estimates important molecular fragments in bait compounds that impart functional significance

Although we train DRIFT by using only the data of the binding affinity between the compound and the protein, the attention layer of DRIFT can identify specific interactions between fragments in the compound and residues in the protein. In natural language processing neural networks, self-attention endows the neural network with the ability to predict the correlation between each word in the sentence. In DRIFT, the attention can help predict the correlation between each compound fragment and protein residue. Thus, we employ DRIFT to further analyze the correlation between fragments in the query compounds and residues in the predicted targets of doxorubicin (Fig. 3B). DRIFT identifies the important constitutive fragments and subsequently evaluates the presence of the identified top-ranked fragments in structurally similar molecules. Our analysis identifies that either –C– or –O– are the most frequent fragments across structurally similar chemical compound pools of any query compound. The third and fourth most-frequent fragments are usually the core structure of the query compound, indicating that they may play significant roles in binding to targets (Fig. 3B). DRIFT ranks the fragments by the attention layer. We compare the profiles of potential targets for two major fragments (fragment 4 and fragment 9 in Fig. S2) in doxorubicin. Fragment 4 and fragment 9 share targets such as tyrosyl-DNA phosphodiesterase 1, thyroid hormone receptor beta-1, and nuclear receptor ROR-gamma. Fragment 4 alone targets telomerase reverse transcriptase and huntingtin, whereas fragment 9 alone targets microtubule-associated protein tau, hypoxia-inducible factor 1 alpha, and three other receptors. Identifying the difference in fragment target profiles can aid in eliminating off-target binding. The highest ranked fragments in a pool of chemically similar molecules can be linked or expanded to generate lead compounds, which is the prime objective of fragment-based drug discovery.

### 2.4. Evaluation of serotonin-based drugs and their target interaction space

**Fig. 4.**
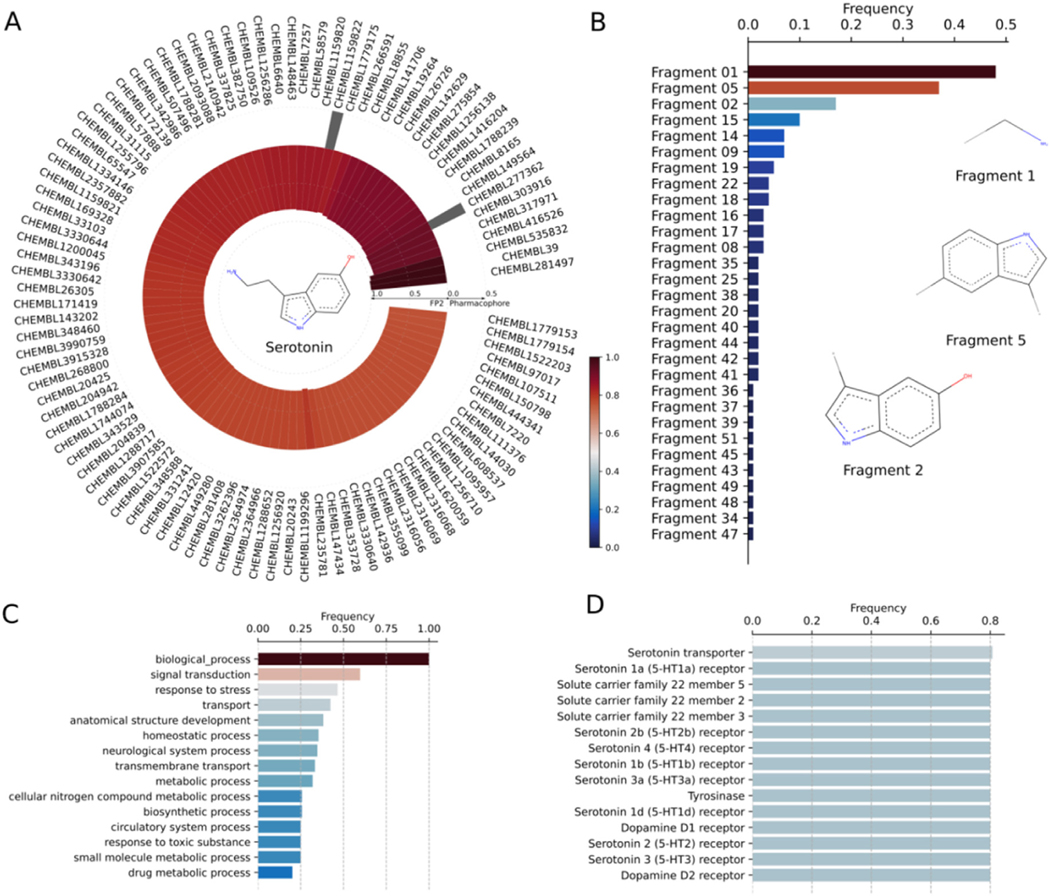
(A) Circular plot depicting the ChEMBL IDs of Serotonin’s bait compounds. 2D structures of bait compounds is included in the center of the circular plot. The outer bars refer to the pharmacophore similarity of compounds and the inner bars refer to the FP2 similarity of compounds. (B) Zipf’s plot showing the rank distribution of chemical fragments of Serotonin with respect to their frequency of occurrence. 2D structures of three most frequently occurring fragments of Serotonin and its bait compounds are shown. (C) Top 15 biological processes or functions executed by the targets of Serotonin and its bait compounds. (D) The list of top 15 proteins estimated to be targeted by Serotonin.

Serotonin (5-hydroxytryptamine) is a small chemical neurotransmitter in the brain that regulates various biological and physiological responses. The secretion of serotonin is important for mood and cognition, and to modulate several psychiatric disorders. Serotonin binds to a group of GPCRs, known as 5-HT receptors or serotonin receptors, in the central and peripheral nervous systems to mediate the neurotransmission. The existence of numerous subtypes of 5-HT receptors throughout the body describes the wide-range of serotonin activity in normal and abnormal states and furnishes a plethora of opportunities for drug development against various psychoneurotic disorders. The discovery or designing of various serotonin-based drugs, those that either suppress or increase the levels of serotonin, as antipsychotics, anxiolytics, and anti-depressants demonstrate the elaborated target space of serotonin. Despite therapeutic modulation activity, the off-target binding of these drugs elicits moderate to severe side effects. The undesired target binding of some antidepressant drugs causes nausea, insomnia, constipation, sexual dysfunction, and weight gain (Beattie and Smith, 2008; de Wit et al., 2005; Stahl et al., 2009). Pergolide, a serotonergic drug, was found to be associated with cardiac fibrosis (Schade et al., 2007; Zanettini et al., 2007). To identify all binding targets of serotonin and serotonin-based drugs and their binding affinity, we perform DRIFT analysis (Fig. 4). We use serotonin’s structure in SDF format as query and employ default DRIFT parameters (Table S1) for the target identification. Our DRIFT approach identifies various classes of annotated serotonin receptors, such as serotonin receptor −1a, 1b, 1d, 2a, 2b, 2c, 6, and 7. In addition, DRIFT also identifies polyspecific organic cation transporters and tyrosinase as potential targets with Kd in the range of nano-to milli-molar concentration (Fig. 4). Finally, we compare the target profiles of serotonin to a structurally similar compound of serotonin, 5-methoxytryptamine (5-MT). As shown in Fig. S5, serotonin receptors are the targets of both serotonin and 5-MT. However, serotonin has been experimentally validated to have many off-target receptors, such as tyrosinase (Yamazaki et al., 2009) and carbonic anhydrase (Isik et al., 2009), and their DRIFT scores are predicted to be as high as those of the serotonin receptors. Although these two targets are also in the list of the predicted targets of 5-MT, their DRIFT scores are lower than those of the serotonin receptors, suggesting that 5-MT has much lower binding affinities with tyrosinase and carbonic anhydrase than with the serotonin receptors; experimentally, tyrosinase and carbonic anhydrase have been identified as the targets of serotonin but not been identified as the targets of 5-MT.

### 2.5. Exploring the target interaction space of β-endorphin opioid neuropeptide

**Fig. 5.**
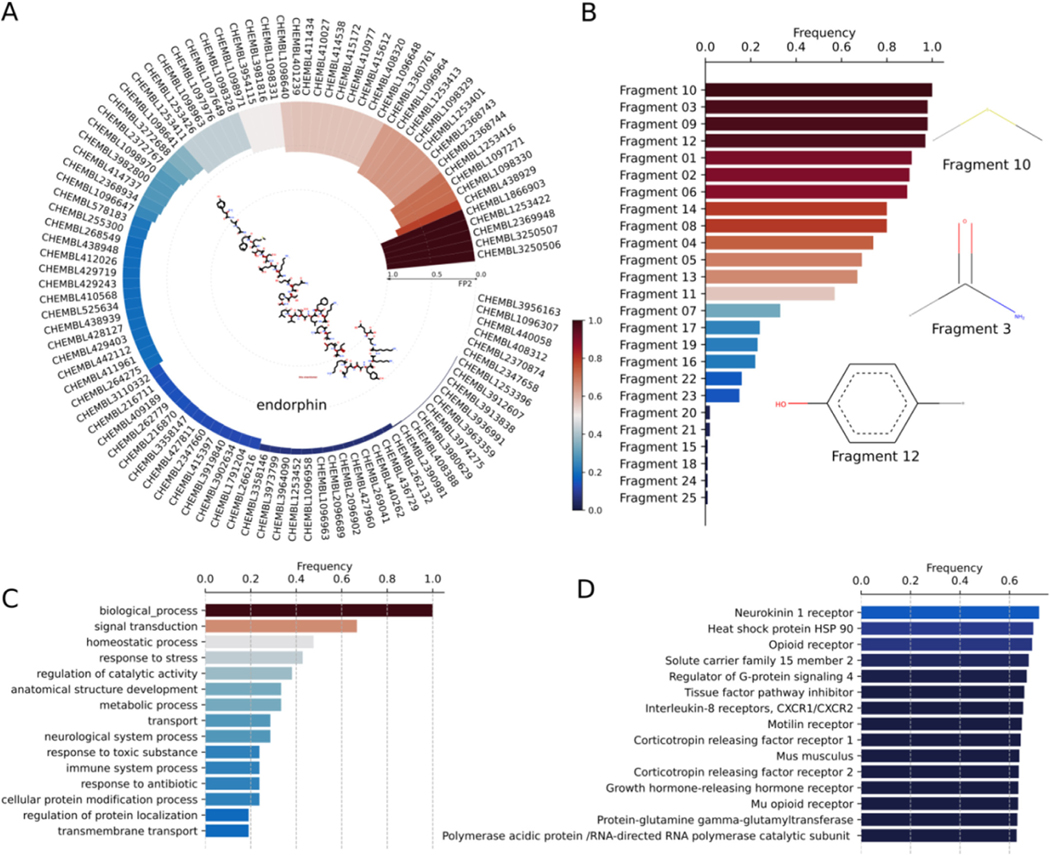
(A) Circular plot depicting the ChEMBL IDs of β-endorphin’s bait compounds. 2D structures of bait compounds is included in the center of the circular plot. The bars refer to the FP2 similarities of compounds. These bait compounds have no pharmacophore similarities because the query compound is too large, and the pharmacophore database only stores compounds with less than 50 atoms. (B) Zipf’s plot showing the rank distribution of chemical fragments of β-endorphin with respect to their frequency of occurrence. 2D structures of three most frequently occurring fragments of β-endorphin and its bait compounds are shown. (C) Top 15 biological processes or functions performed by the targets of β-endorphin and its analogs. (D) The list of top 15 protein targets of β-endorphin as estimated by DRIFT.

Endorphins are opioid neuropeptides that suppress the transmission of pain signals to the brain. Endorphins primarily bind to μ-opioid receptors and synthesized by the pituitary gland and the hypothalamus in vertebrates. Endorphins are classified into three different classes: (a) α-endorphin, (b) β-endorphin, and (c) γ-endorphin. Out of these, β-endorphin is the primary endogenous ligand for μ-opioid receptors and composed of 31 residues with the sequence Tyr-Gly-Gly-Phe-Met-Thr-Ser-Glu-Lys-Ser-Gln-Thr-Pro-Leu-Val-Thr-Leu-Phe-Lys-Asn-Ala-Ile-Ile-Lys-Asn-Ala-Tyr-Lys-Lys-Gly-Glu. β-endorphin modulates stress and pain perception, and maintains homeostasis, excitement, hunger, and reward cognition. Despite the significance of β-endorphin in modulating several biological functions, the target interaction space of β-endorphin is not widely explored. Hence, we employ DRIFT tool to identify potential targets of β-endorphin in entire human proteome (Fig. 5). DRIFT identifies several annotated and new targets for β-endorphin. Specifically, the identification of experimentally known G protein-coupled receptors (GPCRs) such as neurokinin 1 receptor, opioid receptor, corticotropin-releasing factor receptor 1, Regulator of G-protein signaling 4, Motilin receptor, and Interleukin 8 receptors, validate the accuracy of predicted targets (Fig. 5). Apart from the GPCRs, DRIFT identifies tissue factor pathway inhibitor (TFPI), which regulates the extrinsic pathway of blood coagulation (Bajaj et al., 2001; Roberts et al., 2006), as another potential target for β-endorphin. Furthermore, DRIFT predicts heat shock protein 90 (HSP 90) as one of the targets of β-endorphin analogs with IC_50_ = 2 μM. The target-interaction space of β-endorphin may assist in the design of novel drug compounds with minimal side-effect profiles for various disease conditions. Additionally, the therapeutic activity of β-endorphin can be improved by inducing mutations in β-endorphin residues.

### 2.6. DRIFT predicts targets of cannabigerol

Within the plant *Cannabis sativa*, cannabigerol (CBG) is the precursor molecule for the biosynthesis of all the other phytocannabinoids including the two most abundant cannabinoids, Δ^9^-tetrahydrocannabinol (THC) and cannabidiol (CBD) (Nachnani et al., 2021). Because of its role as a precursor, very little CBG is produced by most plants: however, there is a growing interest in the potential pharmacological activities of this molecule in its own right, and high CBG-producing *Cannabis* strains have been developed for this reason (Nachnani et al., 2021). CBG has been shown to interact with common cannabinoid receptors such as CB1 (Cascio et al., 2010; Navarro et al., 2018; Pollastro et al., 2011), CB2 (Cascio et al., 2010; Navarro et al., 2018; Pollastro et al., 2011), TRPV1–4 (De Petrocellis et al., 2011, 2012), TRPA1 (De Petrocellis et al., 2011), and TRPM8 (De Petrocellis et al., 2011). While THC and CBD share a similar structure, the structure of CBG is unique and therefore CBG may interact with different receptors compared to other phytocannabinoids. In fact, CBG has been demonstrated to be an alpha-2 adrenergic agonist, whereas interactions with this receptor have not been reported for other phytocannabinoids. We apply DRIFT to CBG and find that the top six hits from DRIFT reflect known targets of CBG including the cannabinoid receptors and TRP ion channels (Table 1). The top previously unknown hit is GPR55, a known target of CBD and THC that has yet to be shown to bind CBG (Ryberg et al., 2007). The next several targets have not been described to interact with any cannabinoids. Hit ranked #16, the serotonin receptor 5-HT1A, has previously been shown bind both CBD and CBG with CBD acting as an agonist (Rock et al., 2012; Russo et al., 2005) at this receptor, while CBG acts as an antagonist (Cascio et al., 2010; Rock et al., 2011). The remainder of the targets predicted by DRIFT are novel, with the exception of PPARα (D’Aniello et al., 2019) and PPARɣ (Granja et al., 2012). DRIFT not only predicts known targets of CBG with high scores, but it also predicts that this molecule will interact with several previously unknown receptors. These findings will need to be validated; however, based upon the scores and the fact that known targets of CBG have lower scores in this analysis, it is likely that at least some of the novel targets will prove to interact with this understudied cannabinoid.

## 3. Discussion

Identification of target interaction space is essential to designing, developing, or repurposing bioactive molecules with desired pharmacological and physiological properties. Currently, several biochemical and computational approaches are available to predict the targets of small chemical molecules. However, low sensitivity, poor specificity, and high memory/computational requirements hamper the application of these computational biochemical tools for target prediction. Here, we developed a new computational tool, DRIFT, to explore the target interaction space of compounds by combining chemical similarity and machine learning. IN the machine learning process, given a compound-protein pair, DRIFT generates fragments for the compound and then embeds the compound according to its fragments, and DRIFT encodes the protein according to its sequence. DRIFT views the compound as a sequence of fragments, while the protein is composed of a sequence of residues, thus, each fragment or residue is equivalent to a word in a natural language processing (Chowdhury, 2003) task. We apply positional encoding for the embedding of protein residues, but we do not apply positional encoding for the embedding of compound fragments, because the order of residues in protein sequence determines its structure, whereas the order of fragments in a compound is arbitrary. To demonstrate the performance, accuracy, and functionality of DRIFT, we have explored the target interaction space of doxorubicin, serotonin, β-endorphin, and CBG. The target interaction space and bait compound data generated by the DRIFT tool in each test case corroborates experimental findings. Furthermore, the fragmentation and cross-attention approach of DRIFT allows identification of bonds with rich information content and fragments that confer functional activity to the chemical molecule.

We develop the DRIFT platform to accelerate target identification and validation in an economical manner. The DRIFT webserver features: (a) a user-friendly web server, (b) an integrated molecular structure drawer for query compounds, (c) a detailed output layout with data filters to visualize desired target information, (d) automated updating of linked bioactive databases, and (e) accuracy. The DRIFT webserver enables users to explore the proteome-wide target interactions identified from cell-based assays for any set of compounds. DRIFT performs this analysis rapidly (in minutes) and identifies key chemical fragments and protein interactions, aiding in the design and testing of novel therapeutics. The DRIFT webserver allows for efficient analysis of large amounts of target data obtained for annotated bioactive compounds. The annotated targets and the identified fragments of the query compound will allow evaluation and neutralization of off-target interactions by designing new compounds through functional group modification. Incorporating DRIFT in the drug discovery workflow reduces the usage of expensive and time-consuming experimental testing methods during the drug discovery process. Characterizing side effect profiles and potential interacting targets can reduce drug failure rate during clinical studies and expedite the drug discovery process.

## 4. Methods

### 4.1 Similarity searching through pharmacophore and FP2 fingerprints

The structural similarity between two molecules can be visually identified by locating common substructures, structural motifs, or functional groups. However, to precisely discern structural similarities between two ligands, similarity matching algorithms based on the “chemical similarity principle” (Cereto-Massagué et al., 2015; Johnson and Maggiora, 1990; Maggiora et al., 2014) are essential. The chemical similarity principle (Cereto-Massagué et al., 2015; Johnson and Maggiora, 1990; Maggiora et al., 2014) states that if two chemicals share common structural properties, then they tend to bind similar protein targets and exhibit similar bioactivities. We posit that the targets of a query compound can be identified from that of known drugs and metabolites which share structural components with a query compound. In the past decade, numerous computational algorithms (Daina et al., 2019; Dunkel et al., 2008; Gong et al., 2013; Keiser et al., 2007; Lo et al., 2015; Wang et al., 2013) have been published for chemical similarity scoring and searching. In this work, we utilize pharmacophore and molecular fingerprints for compound similarity searching and subsequent estimation of the compound’s target interaction space. A pharmacophore is an abstract description of molecular features that are necessary for molecular recognition of a ligand by a biological macromolecule. A molecular fingerprint is a hash function that summarizes molecular features such as composition, connectivity, and stereochemistry. We utilized Pharmer (Koes and Camacho, 2011), a computational approach to pharmacophore search that scales with the breadth and complexity of the query, rather than the size of the database, for pharmacophore searching. Initially, we downloaded structure files of all ChEMBL chemical compounds and employed Pharmer to create Bloom fingerprints – a tetrahedral (3D) pharmacophore fingerprint for fast pharmacophore matching. Simultaneously, we used the OpenBabel package to generate the two-dimensional fingerprint (FP2) for the query compound(O’Boyle et al., 2011). We imposed the following conditions while generating an FP2 fingerprint: (i) The molecular fragment shall contain at most seven atoms. (ii) Single atom fragments of C, O, and N shall be ignored. (iii) Duplicate entries for each generated fragment shall be omitted. (iv) Molecular fragment shall terminate when the constituent atoms form ring structures. We evaluated the details of atoms, bonding, and presence of cyclic ring structures for each of the generated fragments and assigned a hash number. We formulated a 1024-bit vector from the generated hash numbers as the FP2 fingerprint for the query compound. Similarly, we generated FP2 fingerprints for annotated chemical compounds from the ChEMBL (version 25) (Gaulton et al., 2011), Zinc (Irwin et al., 2012), HMDB (Human Metabolome Database) (Wishart et al., 2018), and BindingDB (Wishart et al., 2017) databases. Subsequently, we built a *fastsearch* index using OpenBabel (O’Boyle et al., 2011) to store the datasets of FP2 fingerprints from the curated bioactive databases for rapid similarity search and comparisons. The similarity of FP2 fingerprints is determined by the Tanimoto coefficient ($T_{c}$). The $T_{c}$ score is a widely used metric for quantifying chemical similarity between two ligand molecules. $T_{c}$ compares two molecular fingerprints and evaluates the fraction of common structural moieties between two compounds A and B using the expression: $T_{c}(A,B)=\frac{c}{a+b-c}$ where c denotes common bits in two fingerprints, a and b denote bits from fingerprint sets for A and B. The value of $T_{c}$ ranges from ‘0’ to ‘1’, where $T_{c}=0$ represents highly dissimilar compounds and $T_{c}=1$ represents identical compounds. Two chemical compounds are generally considered structurally similar if $T_{c}\geq 0.85$. The parameters that are used for similarity searching are listed in Table S1.

### 4.2. Compound-protein interaction prediction

We constructed a sequence convolution- and graph convolution based neural network to predict the binding affinity between a molecular compound and a protein. As input to the network, we encode (i) the compound’s 2D structure (SMILES (Weininger, 1988) or INCHI (Stein et al., 2003) code) and (ii) the protein’s amino acid sequence. The network first encodes the protein using a BLOSUM62 matrix (Eddy, 2004). For simplicity, the BLOSUM62 features of non-standard amino acids are initialized to zero. Then, the BLOSUM62 protein features are processed through three 1D convolution layers. Given the SMILES representation of a compound, the network first employs rdkit (Landrum, 2013) to represent it by a graph (*N, V, E*), where *N* is the number of nodes, *V* is the feature vector of each atom, and *E* is the feature vector of each bond. The feature of each atom is the concatenation of the one-hot encoding of atom type, number of bonds, bond type, mass, and charge vectors, whereas the feature of each bond is the bond order. This graph representation of the compound is then subjected to two graph convolution layers. The protein features and the compound features are then subjected to 5 feature-wise fully connected layers, separately. We implemented this network using TensorFlow 2.3.1 (Abadi et al., 2016) and Sonnet 2. The GNN was implemented using the *graph_nets* (Scarselli et al., 2008) library.

### 4.3. Training

The objective is to minimize the mean squared error of the predicted binding affinity and the experimental binding affinity. During the training process, we used a mini-batch of 8 and the Adam stochastic optimizer (Kingma and Ba, 2014) to optimize the model parameters. The benchmark datasets included the PDBbind dataset (Liu et al., 2015), the Davis dataset (Davis et al., 2011), and the Metz dataset (Metz et al., 2011). The PDBbind dataset is a high-quality set of protein-ligand complexes with available structural data and corresponding binding affinities. The PDBbind dataset is compiled from the PDBbind database (version 2018, the general set), which contains a high-quality set of protein-ligand complexes with available structural data and corresponding binding affinities. Each complex was provided with an affinity value of a certain measurement type. The Davis dataset contains interactions of 72 kinase inhibitors with 442 kinases covering >80% of the human catalytic protein kinome. The Davis dataset was obtained from the Supplementary Information of Davis et al. (2011). The Metz dataset was obtained from the Supplementary Information of Metz et al. (2011). The PDBbind dataset contains 2509 small molecules and 10251 proteins, with a total of 13,311 interactions. On average, each small molecule corresponds to 5.3 interactions and each protein corresponds to 1.3 interactions. The Davis dataset contains 68 small molecules and 379 proteins, with a total of 9125 interactions. On average, each small molecule corresponds to 134.2 interactions, and each protein corresponds to 24.1 interactions. The Metz dataset contains 1471 small molecules and 172 proteins, with a total of 35,307 interactions. On average, each small molecule corresponds to 24.0 interactions, and each protein corresponds to 207.7 interactions. We divided each dataset into a training set and a test set at a ratio of 8 to 2.

### 4.4. DRIFT web server to extend and streamline the application of chemical similarity search for a wide range of chemical compounds

To facilitate the use of our computational strategy, based on a chemical similarity search for compound target prediction, we developed a user-friendly web server with broad applications. We implemented an internal compound searching system to accept various types of inputs including uploading the query compound in SDF or SMILES format, providing a database ID, or entering the name of the query compound. The submitted query compound along with related parameters are stored in a queue. Based on the search parameters chosen by the user, DRIFT searches the CHEMBL (Gaulton et al., 2011), Zinc (Irwin et al., 2012), HMDB (Wishart et al., 2018), and BindingDB (Wishart et al., 2017) databases. Subsequently, DRIFT retrieves the associated targets of similar annotated compounds from these curated databases (Fig. 1). The obtained target data are then queried against the Gene Ontology database for the functions and involved processes of the targets. Finally, the DRIFT server compiles the results page, returning chemically similar bait compounds, associated targets, biological functions of targets, and involved biological processes (Fig. 1). The front-end of the DRIFT website is developed on the vue.js framework. The three major vue modules that undertake most of the functions of the website are: ‘submit’, ‘queue’, and ‘task’. The ‘submit’ module provide a user-friendly interface to submit the query compound. The user can search, upload, or directly draw the structure of the compound using Marvin JS sketcher (www.chemaxon.com, version 19.27.0) (Fig. S3). The ‘queue’ module notifies the status of submitted tasks. Successful completion of a task generates output in five segments: ‘compounds’, ‘targets’, ‘functions’, ‘processes’, and ‘fragments’. The ‘compounds’ section contains all chemically similar bait compounds within a chosen Tc threshold. The ‘targets’ section includes all the annotated targets of the bait compounds. The ‘functions’ section summarizes all biological activities of the annotated targets. The ‘processes’ section shows all the biological or metabolic processes in which the annotated targets are involved. Finally, the ‘fragments’ section emphasize high-information-content bonds or fragments that confer functional activity to query the molecule along with the identified bait compounds. The backend of DRIFT is written in the PHP language. The website is hosted on a Linux server and managed by Apache. A MySQL database is used to store most of the information, including the users and tasks. Finally, to help users select targets with a low probability of finding false positives, we adopt a strategy to assign confidence to each predicted target. If a protein-ligand interaction has been validated in more than one assays, then this interaction is assigned high confidence; otherwise, it is assigned low confidence. Since the probability that two or more different assays all yield false positives is low, this strategy can improve the confidence of the predictions.

### 4.5. Compound fragmentation approach

A compound structure file, irrespective of the format, is initially converted to ‘mol2’ format through OpenBabel. A mol2 file includes two main sections: (a) atom section and (b) bond section. Initially, our program retrieves bond information (Fig. S4) from the bond section and annotates cyclic ring structures (Fig. S4), if any, in the compound. Subsequently, bonds in identified cyclic ring structures are labeled as ‘non-rotatable’ (Fig. S4). Next, high order bonds also labeled as ‘non-rotatable’. Finally, we employ a clustering algorithm to generate fragments that satisfy the following two conditions: (i) atoms in each fragment connect by non-rotatable bonds; (ii) atoms between different fragments connect by rotatable bonds. The workflow of the clustering algorithm is as follows: Initially, we treat each atom of the chemical compound as an individual cluster. Then, we combine the atoms in different clusters iteratively into a single cluster if the clusters are connected by non-rotatable bonds (Fig. S4). Finally, we assign fragments to each segregated cluster of connected atoms (Fig. S4).

### 4.6. Evaluation

We compile two datasets for the evaluation of DRIFT in terms of similar compound searching (DS-I) and drug targets identification (DS-II). These datasets are compiled from ChEMBL v25 database (Mendez et al., 2019). We select assays with reported kd values (kd > 0) and associated target type – “SINGLE PROTEIN”. We extract all associated targets and compounds, attaining the target sequence and compound’s SMILES code. Altogether, we have 82910 compound-protein interactions involving 1700 human proteins and 24,412 small molecules. We compose DS-I by selecting all proteins that have at least 20 associated compounds. DS-I has 109 proteins and 6479 compounds. We compose DS-II by selecting all compounds that have at least 5 associated targets. DS-II has 110 compounds and 2323 proteins. The two datasets are presented in the supporting information (SI) data. When evaluating the performance of DRIFT, we used the default parameters (Table S1). When evaluating the performance of ChemMapper, we selected the default parameters for the similarity method (SHAFTS) and the similarity threshold (1.2). To search for as many targets as possible, we selected BindingDB (412154 interactions) as the database. When evaluating the performance of SwissTargetPrediction, we selected the default parameters.

## Supplementary Material

spreadsheet

Word document

## Figures and Tables

**Table 1:** DRIFT predictions of pharmacological targets for cannabigerol (CBG). The structure of CBG was submitted for DRIFT analysis without a priori guidance. The top 16 “hits are presented. It is remarkable that seven known targets of CBG were identified. Moreover, while the 5HT-1a receptor has a lower score, it is reported to have a high affinity for CBG (50 nM).

Target	Score	Known CBG Target	Function
Cannabinoid receptor 1 (CB1)	0.79	Yes; 1.05 μM Ki	Agonist
Transient receptor potential catio channel subfamily M member 8 (TRPM8)	0.73	Yes; 0.16 μM Ki	Antagonist
Canabinoid receptor 2 (CB2)	0.73	Yes; 1.23 μM Ki	Agonist
Transient receptor potential cation channel subfamily V member 2 (TRPV2)	0.71	Yes; 1.72 μM Ki	Agonist
Vanilloid receptor	0.69	Yes; 1.3 μM Ki	Agonist
Transient receptor potential cation channel subfamily A member 1 (TRPA1)	0.69	Yes; 0.7 μM Ki	Agonist
G-protein coupled receptor 55 (GPR55)	0.66	No	Unknown
Voltage-gated L-type calcium channel alpha- 1C subunit	0.64	No	Unknown
Cytochrome p450 3A11	0.63	No	Unknown
Cytochrome p450 1A1	0.62	No	Unknown
Arachidonate 15-lipoxygenase	0.62	No	Unknown
Arachidonate 12-lipoxygenase	0.61	No	Unknown
Anandamide amidohydrolase	0.60	No	Unknown
Cholesteryl ester transfer protein	0.60	No	Unknown
Nuclear receptor ROR-gamma	0.60	No	Unknown
Serotonin 1a receptor (5-HT1a)	0.58	Yes; 0.05 μM Ki	Antagonist

## Data Availability

Table S1 and Figs. S1–S5 are in the Supporting Information (SI) file. The dataset files and test result files are in the Supplementary Data. The DRIFT webserver: http://Drift.Dokhlab.org. DRIFT source codes: https://bitbucket.org/dokhlab/drift-daemon.
