# Supplementary material for: Whole proteome mapping of compound-protein interactions
Source: Curr Res Chem Biol. Author manuscript; Available in PMC 2023 Dec 20. (PMC10732549; doi:10.1016/j.crchbi.2022.100035)
Supplement: spreadsheet [file NIHMS1905629-supplement-spreadsheet.docx]

**SUPPORTING INFORMATION**

**Whole Proteome Mapping of Compound-Protein Interactions**

Venkat R. Chirasani^1,2,#^, Jian Wang^1,#^, Congzhou Sha^1^, Wesley Raup-Konsavage^1^, Kent Vrana^1^,

and Nikolay V. Dokholyan^1,3,4,5,*^

Table S1. The list of DRIFT parameters for target identification.

| Parameter | Value |
| --- | --- |
| Task title  Query compound  Database  FP2 Similarity Cutoff  Compound Similarity Metrics  Number of bait compounds | (Optional)  SDF or SMILES format  CHEMBL, Zinc, Metabolites, and BindingDB  0.8  FP2, Pharmacophore  100 |


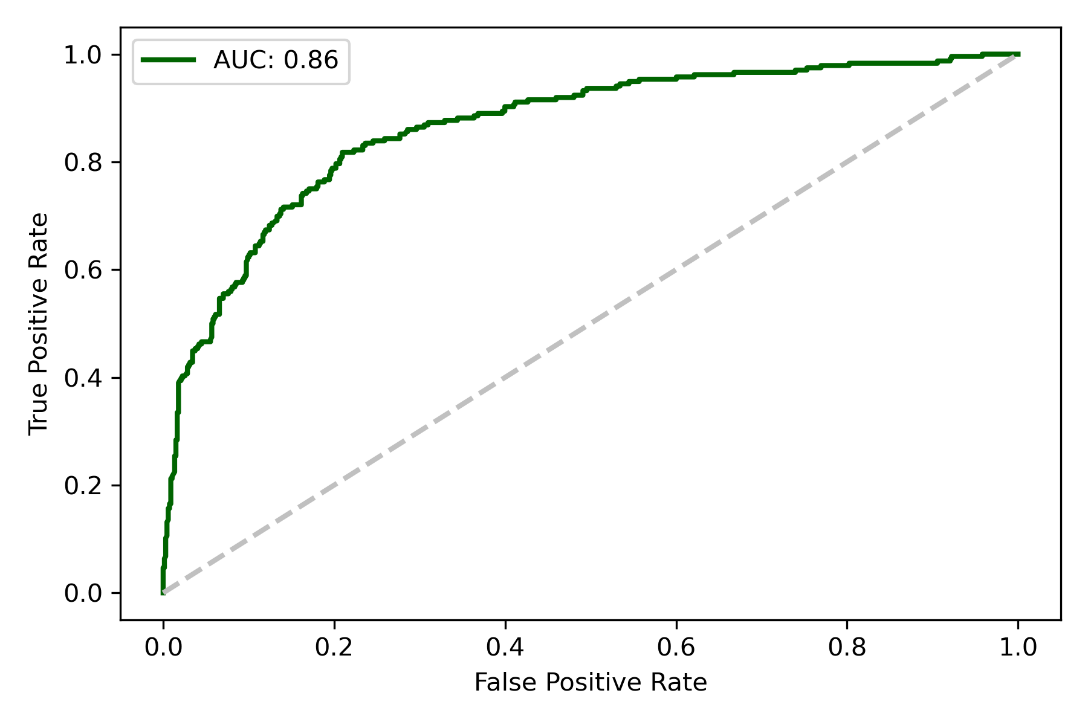


**Figure S1. The ROC curve of the evaluation of DRIFT on the Davis data set.** The AUC of the prediction is 0.86.


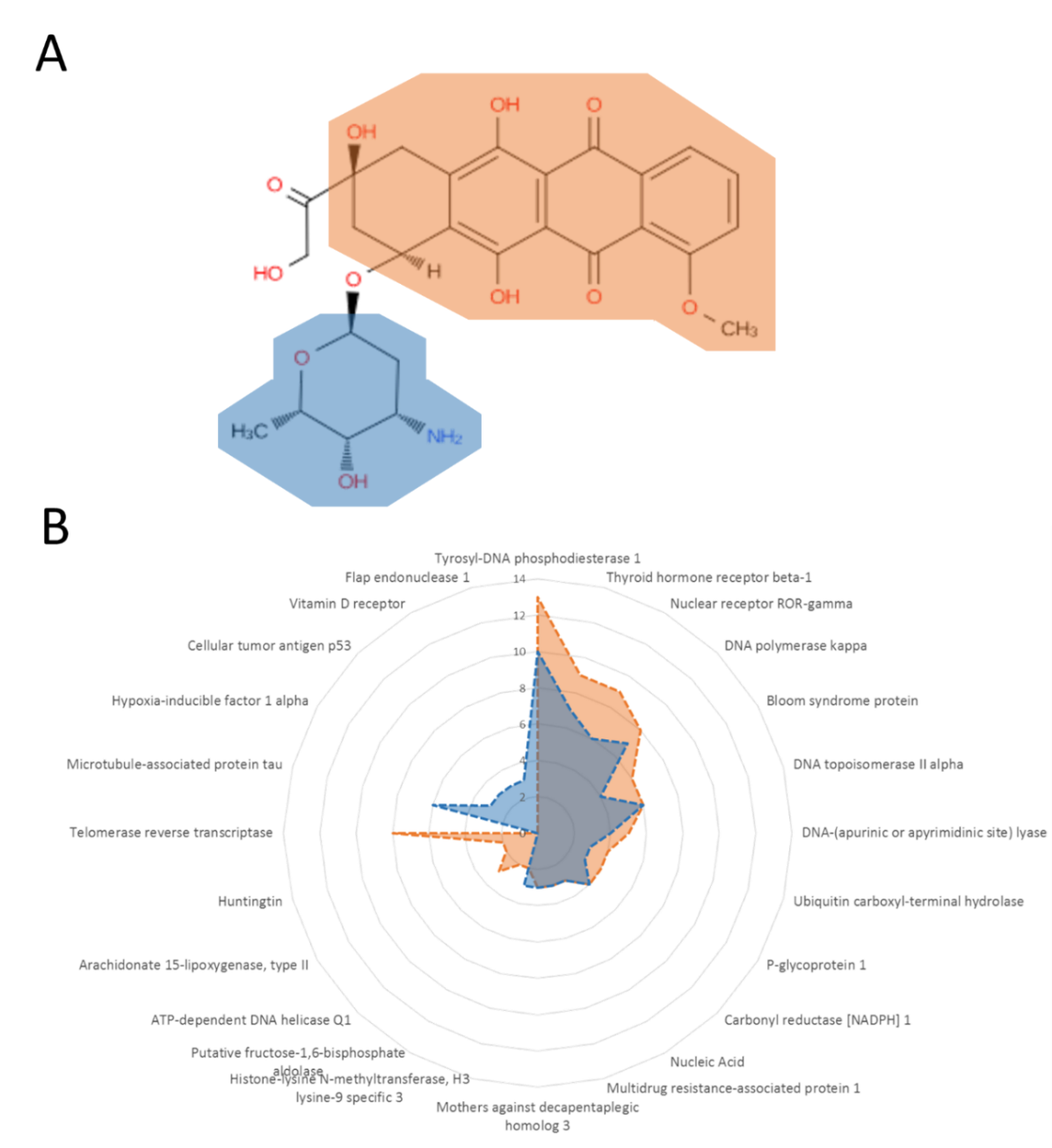


Figure S2. Comparison of the targets of two major fragments in doxorubicin. (A) The two major fragments in doxorubicin. The red area refers to Fragment 4 and the blue are referring to Fragment 9. (B) The radar plot of the profiles of potential targets of Fragment 4 and Fragment 9.


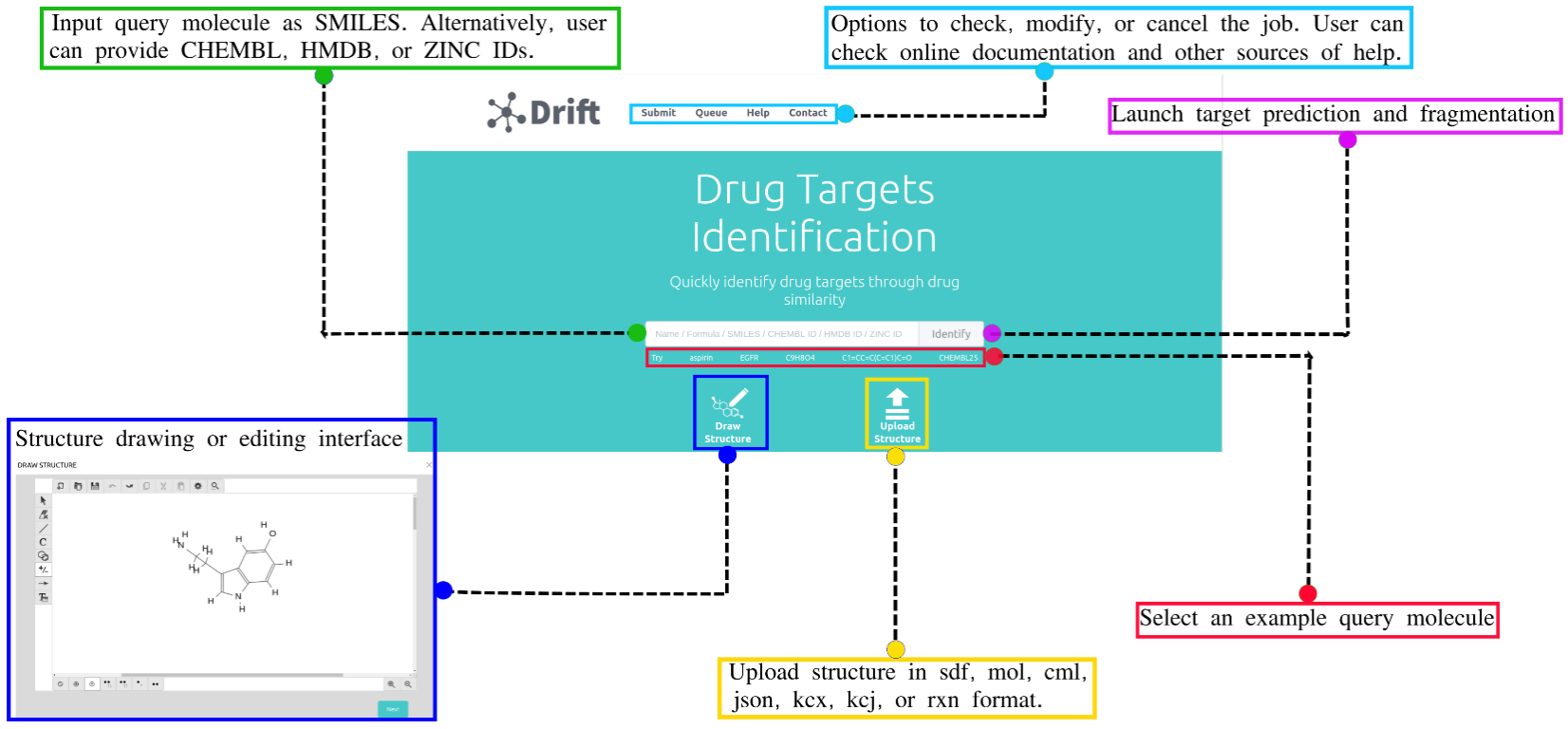


Figure S3. Outline of Drift target prediction web interface. Each option available on Drift webserver is explained in zoomed-out boxes. The query molecule can be either uploaded in various formats or can be drawn or edited using Kekule.js Render. Some specific examples are provided beneath the input field to assist users in understanding query structure format and other search parameters. Once the query molecule is inputted, clicking the ‘Identify’ button will outline various search parameters such as intended target databases, Tanimoto coefficient cut-off, and the number of annotated targets to explore.


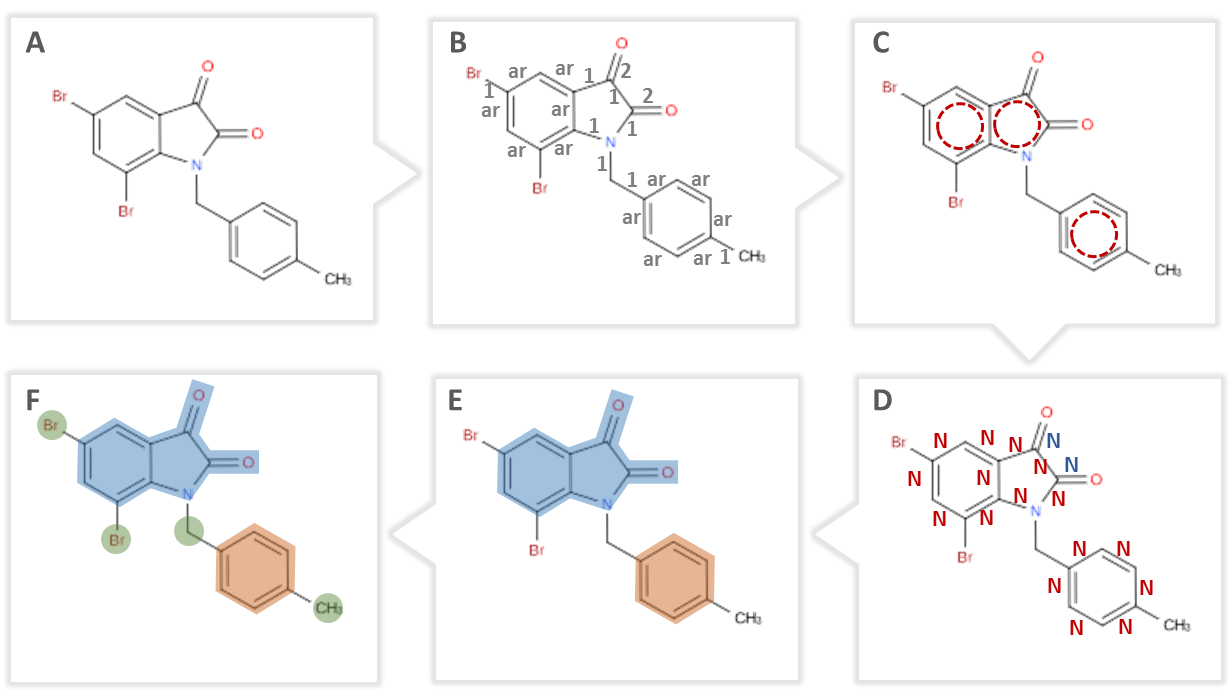


Figure S4. Workflow of the fragmentation algorithm. (A) Input compound. (B) Calculation of the bond orders. (C) Identification of rings in the compound. (D) Labelling ring bonds and high order bonds as “non-rotatable”. Bonds that are labeld by “N” are identified as non-rotatable. (E) Connecting atoms by non-rotatable bonds. Blue and red regions cover the atoms that are connected by non-rotatable bonds, respectively. (F) Fragmentation of a compound based on connected atom groups.


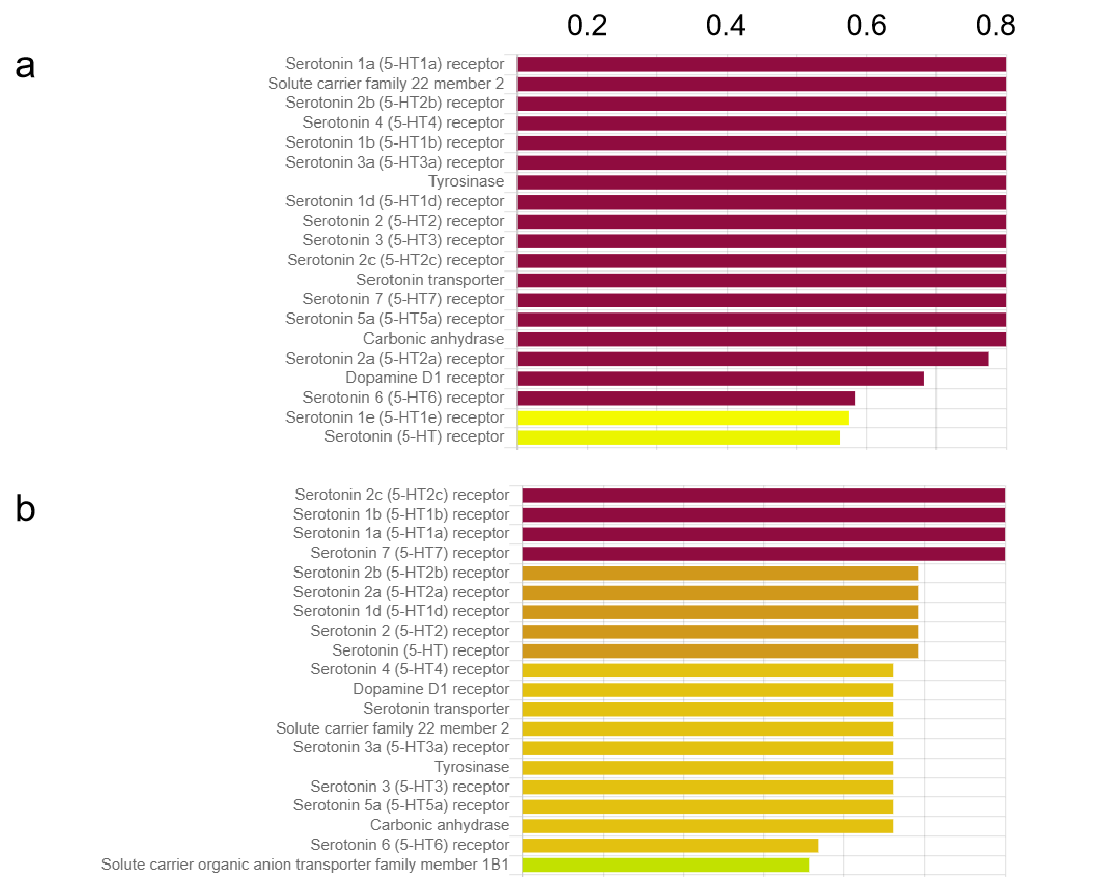


Figure S5. The comparison of the target profiles of serotonin to that of 5-MT (5-Methoxytryptamine). (a) The top 20 targets that have the highest predicted binding affinities with the serotonin. (b) The top 20 targets that have the highest predicted binding affinities with 5-MT.
